# Umbu Fruit Peel as Source of Antioxidant, Antimicrobial and α-Amylase Inhibitor Compounds

**DOI:** 10.3390/molecules27020410

**Published:** 2022-01-09

**Authors:** Leilson de Oliveira Ribeiro, Beatriz Pereira de Freitas, Carolline Margot Albanez Lorentino, Heloisa Freire Frota, André Luis Souza dos Santos, Davyson de Lima Moreira, Bruno Sérgio do Amaral, Eliane Przytyk Jung, Claudete Norie Kunigami

**Affiliations:** 1 Laboratory of Organic and Inorganic Chemical Analysis, National Institute of Technology, Rio de Janeiro 20081-312, Brazil; eliane.jung@int.gov.br (E.P.J.); claudete.kunigami@int.gov.br (C.N.K.); 2 Faculty of Chemical Engineering, Federal University of Fluminense, Niterói 24210-240, Brazil; beatrizzfreitas11@gmail.com; 3 Laboratory for Advanced Studies of Emerging and Resistant Microorganisms, Federal University of Rio de Janeiro, Rio de Janeiro 21941-902, Brazil; carolline.margot@gmail.com (C.M.A.L.); heloisafreiref@gmail.com (H.F.F.); andre@micro.ufrj.br (A.L.S.d.S.); 4 Laboratory of Natural Products, Rio de Janeiro Botanical Garden Research Institute, Rio de Janeiro 22460-030, Brazil; davysonmoreira@jbrj.gov.br; 5 Federal Institute of Education, Science and Technology of São Paulo, São Paulo 05110-000, Brazil; bruno.sergio90@gmail.com

**Keywords:** *Spondias tuberosa*, umbu waste, extraction optimization, mass spectrometry, enzyme activity, antibacterial activity

## Abstract

Herein, the extraction of bioactive compounds from umbu fruit peel was optimized using thermal-assisted solid–liquid extraction. In parallel, antioxidant, antimicrobial, and inhibitory effects against α-amylase of optimized extract were also evaluated. The combination of operational conditions including the temperature (32–74 °C), ethanol concentration (13–97%), and solid/liquid ratio (1:10–1:60; *w/v*) was employed using a rotational central composite design for optimization. The extracts were evaluated for total phenolic compounds (TPC), total flavonoid compounds (TFC) and antioxidant capacity by ABTS^•+^, DPPH^•^ and FRAP assays. The bioactive profile of the optimized extract was obtained by ultra-performance liquid chromatography coupled to quadrupole/time-of-flight mass spectrometry in electrospray ionization in both negative and positive modes. The statistically evaluated results showed that the optimal operational conditions for the recovery of bioactive compounds from umbu fruit peel included 74 °C, 37% ethanol, and a solid–liquid ratio of 1:38. Under these conditions, the obtained values were 1985 mg GAE/100 g, 1364 mg RE/100 g, 122 µmol TE/g, 174 µmol/TE g and 468 µmol Fe^2+^/g for TPC, TFC, ABTS^•+^, DPPH^•^, and FRAP assays, respectively. In addition, the optimized extract was effective against Gram-positive and Gram-negative bacteria (MBC ranged from 0.060 to 0.24 mg GAE/mL), as well as it was effective to inhibit α-amylase (IC_50_ value of 0.076 mg GAE/mL). The optimized extract showed to be mainly constituted by phenolic acids and flavonoids.

## 1. Introduction

Native fruits from Brazil have received attention in recent years for being sources of compounds of great technological and health interest. Fruits such as umbu, camu-camu and juçara are sources of vitamin C and phenolic compounds, for example [1,2]. Umbu fruit from Brazilian semi-arid regions contains bioactive compounds such as rutin, quercetin, carotenoids, and vitamin C, as reported by Ribeiro et al. [3]. This rich composition confers relevant antioxidant potential to fruit. In addition to being a fruit rich in bioactive compounds, umbu fruit plays an important socio-economic role, since it provides and increases the income of small and medium producers from the semi-arid region of Brazil. It is estimated that 7765 tons of this fruit were produced in 2018 [4], also being marketed as frozen pulp [5].

The processing of fruits generates a large volume of waste, mainly composed of peels, seeds and stones. Nowadays, it is well-known that waste from fruit processing may be rich in compounds with high added value. Thus, its use for the recovery of antioxidant and/or colorant compounds has been evaluated [6]. Grape pomace, for example, has been extensively evaluated for this purpose mainly due to its antioxidant potential, which increases antioxidant capacity of developed products and improves their shelf-life [7].

Considering umbu fruit, the peel and seed have already been evaluated for composition in macro and micronutrients and bioactive compounds. Ribeiro et al. [3] reported that the fruit peel presented contents of 1775 mg/100 g and 2751 µg/100 g for total phenolic compounds and total carotenoids, respectively, flavonoids such as rutin and quercetin being identified. β-carotene, zeinoxanthin, and β-cryptoxanthin were highlighted among the carotenoids. The fruit seed was evaluated by Dias et al. [8]. According to those authors, the seed oil was composed of palmitic, stearic, oleic, linoleic and linolenic fatty acids, with high content of unsaturated fatty acids (70–73%). The authors also pointed out that obtained extracts of seeds were rich in phenolic compounds. Omena et al. [9] reported that the umbu fruit fractions, pulp, peel and seed, did not present cytotoxicity in assays using sheep corneal epithelial cells. In addition, phytochemical screening showed the presence of phenols, tannins, anthraquinones, anthrones, coumarins, triterpenoids and steroids in fruit peels extracted using 95% hydroethanolic solution (qualitative assays). The antioxidant activity of this extract was also evaluated using a peroxyl radical-mediated lipid peroxidation membrane model, being observed that umbu fruit peel and seed extracts provided more than 95% protection of the membrane for 15 min. These results were considered better than those obtained by positive controls (Trolox, vitamin C and resveratrol). Cangussu et al. [10] evaluated the potential of peel flours of mature and semi-mature umbu fruit as a source of bioactive compounds, highlighting the presence of trigonelline, an alkaloid with bioactive activities. The authors also evaluated the bioaccessibility of total extractable phenolics, flavonoids, and tannins of umbu fruit peel flour, suggesting that umbu fruit peel flour can be used in food products to replace other flours with lower nutritional and functional values. In this way, data demonstrate the potential of the processing waste of umbu fruit to obtain new products and/or bioproducts. Despite that, to the best of our knowledge, the optimal conditions for the recovery of bioactive compounds from fruit peel have not yet been optimized in order to provide technological approach to obtain a bioactive compound-rich extract with potential application by food and cosmetic industries, as in the following examples. An extract of siriguela fruit peel was used as an active ingredient in the formulation of a sunscreen. Silva et al. [11] reported that the extract composed of dicaffeoylglucose, hexahydroxydiphenoyl-galloyl-glucose, galloyl-bis-hexahydroxydiphenoyl-glucose, rutin, and quercetin (phenolic compounds) promoted protection against UVB ray in a sunscreen formulation at 30% of extract. Extracts of fruit peels also have been used to enrich food films and coatings, since bioactive compounds can exhibit antimicrobial action, which help maintain the postharvest quality of fruits as reported by Gull et al. [12]. Their results showed that apricot fruit treated with nanochitosan coating added with 1% of pomegranate peel extract significantly reduced decay percentage, weight loss, effectively retained antioxidant activity, ascorbic acid, kept titratable acidity and firmness at a higher level than untreated fruit, as well as significantly inhibited total psychrophilic bacterial count, yeast and mold count during storage at 4 °C for 30 days.

Thus, the present work aimed to optimize the extraction of bioactive compounds from the umbu fruit peel and evaluate phytochemical profile of the optimized extract by UPLC-qTOF/MS (ultra-performance liquid chromatography coupled to quadrupole/time-of-flight mass spectrometry). Additionally, the antioxidant effect was evaluated, as well as the effect of the optimized extract against clinically relevant microorganisms and enzymatic activity of α-amylase.

## 2. Results and Discussion

### 2.1. Effect of Independent Variables

It was observed that umbu fruit peel provided antioxidant extracts by different assays as well as being rich in phenolic compounds, as summarized in Table 1. The TPC content changed from 525 mg/100 g to 1986 mg/100 g, showing the strong influence of the extraction temperature, extractive solution, and solid–liquid ratio factors. The highest value for this response was obtained when higher extraction temperatures were employed. This pattern was also observed for TFC content, whose maximum value was 1513 mg/100 g. Our results were superior to data reported by Ribeiro et al. [3], who found 1775 mg/100 g of TPC for umbu fruit peel extracted using 70% acetone (analytical extraction). In addition, those authors reported the presence of quercetin and rutin in this fraction of the fruit. It corroborates the use of TFC content as a response in our extraction study.

In relation to the antioxidant capacity of the extracts of umbu fruit peel it was registered for ABTS^•+^, DPPH^•^ and FRAP assays that this potential increased 12, 3.3 and 4.2 times, respectively. It corroborates those operational conditions which have great influence on antioxidant capacity, as also observed for TPC and TFC contents. Furthermore, the results corroborate that the interaction compound-radical is different, being, therefore, relevant using various assays for evaluation of the antioxidant capacity of plant samples. It is important to emphasize that antioxidant potential observed in the umbu fruit peel is due to the presence of bioactive compounds such as phenolics [13] and their recovery is an alternative to add value to fruit agro-chain, since peels are discarded after depulping.

The highest value found for ABTS^•+^ response in our work (109 µmol Trolox/g) was close to data reported by Ribeiro et al. [3], who evaluated different fractions of umbu fruit (143 µmol Trolox/g). In the study performed by those authors, the fruit peel was submitted to successive extractions with 50% methanol and 70% acetone (analytical extraction), which improves the recovery of bioactive compounds. Thus, they have reported a higher value of antioxidant capacity. However, these solvents are toxic, which can reduce the potential for their further application, mainly in the food industry.

**Figure 1:**
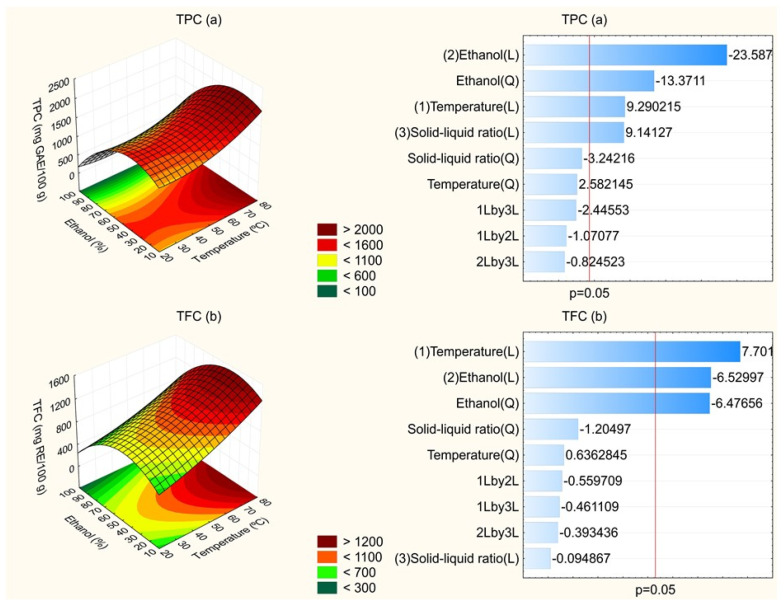
Effect of the independent variables on the total phenolic compounds (TPC) (**a**), total flavonoid compounds (TFC) (**b**).

**Figure 2:**
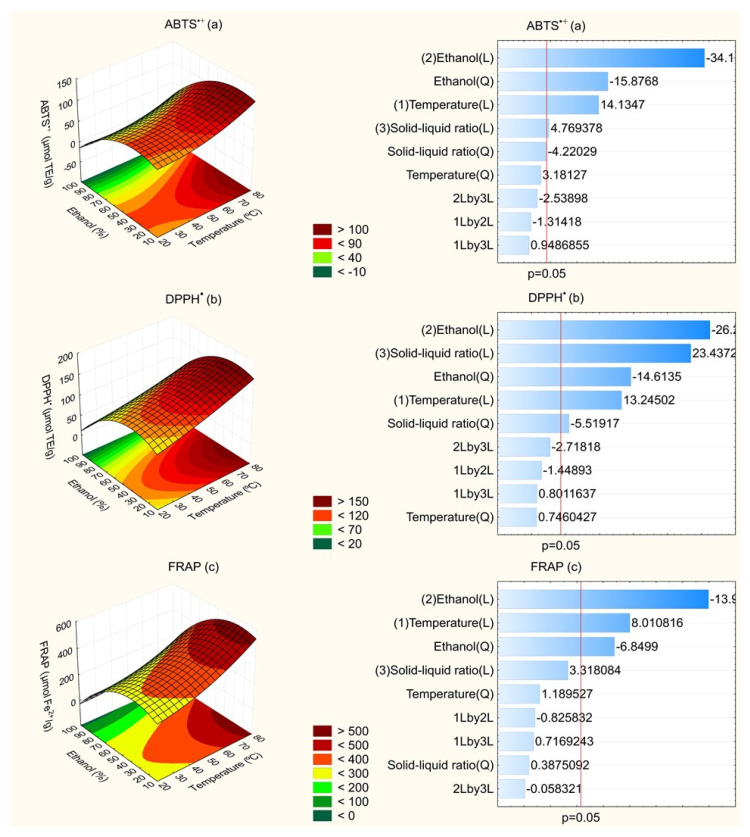
Effect of the independent variables on the antioxidant capacity by ABTS^•+^ (**a**), DPPH^•^ (**b**) and FRAP (**c**) assays.

Regarding statistical analysis, all models were significant for predicting the pattern of the responses in relation to independent variables, since the calculated F-values were higher than listed F-values (F_9,7_ = 3.68) at *p* = 0.05. For TPC, TFC, ABTS^•+^, DPPH^•^ and FRAP responses, calculated F-values were 7.74, 7.27, 16.34, 18.38 and 7.08, respectively. In addition, it is worth emphasizing that lack of fit was non-significant since it presented *p*-values lower than 0.05 and calculated F-values lower than the listed F-value for all responses. The R^2^ values of the fitted models were 0.91, 0.90, 0.95, 0.96 and 0.91 for TPC, TFC, ABTS^•+^, DPPH^•^ and FRAP responses, respectively, showing that the models explained, at least, 90% of the data variability obtained from this experimental design. Therefore, the response surfaces were constructed to relate independent variables and responses. Figure 1 and Figure 2 show the effect of temperature and ethanol concentration, with the solid–liquid ratio fixed at 1:35, on the TPC and TFC contents and antioxidant capacity measured by ABTS^•+^, DPPH^•^ and FRAP assays. The solid–liquid ratio was fixed at 1:35 because it had lower influence on results, as can be seen in Pareto charts (Figure 1 and Figure 2), except for antioxidant capacity by DPPH^•^ assay.

By means of the Pareto Chart (Figure 1a), it is possible to note that ethanol concentration had a higher influence on the TPC content of extracts. The linear effect presented a significant and negative value at *p* = 0.05. Additionally, it was observed that the quadratic effect of this factor was significant. It corroborates that there is a maximum value of ethanol concentration, which promotes higher attainment of phenolic compounds. From this value, there is lower recovery of these compounds. In addition, the linear effects of temperature and solid–liquid ratio were significant and positive. In this way, there is higher recovery of phenolic compounds from umbu fruit peel from increase in these parameters. Through response surface (Figure 1a), it is registered that using temperatures higher than 60 °C and ethanol solution between 20 and 50%, high TPC content was obtained.

It was observed by the TFC content that the positive and linear effect of temperature was that with a higher influence on recovery of flavonoid compounds followed by the linear and quadratic effect of ethanol concentration, which were both negative (*p* < 0.05) (Figure 1b). Thus, as it was cited above, the temperature increases the recovery of flavonoid compounds from fruit peel. For ethanol concentration, there is a limit for this pattern since that quadratic effect was also significant. From the response surface, it is possible to observe that, at zones in intense red, there was higher recovery of flavonoids of umbu fruit peel, which comprises temperature between 70 and 80 °C and ethanol concentration between 20 and 60% (Figure 1b). However, it is important to stress that the boiling point of ethanol is 78.2 °C; therefore, it is not adequate to exceed it. Furthermore, the elevation of temperature can raise the process cost.

The antioxidant capacity by ABTS^•+^ assay had the same behavior observed for TPC content in relation to influence of independent variables. Ethanol concentration exerted a negative effect on this response, being inversely proportional to antioxidant potential of extract, in other words, high ethanol concentrations reduced the antioxidant capacity of extracts by recovering less antioxidant compounds. Temperature above 60 °C and ethanol concentration below 50% comprise the range in the intense red zone with higher antioxidant capacity from this assay (Figure 2a).

By DPPH^•^ assay, the factors with a significant influence on this response were the linear effect of ethanol concentration, the linear effect of solid–liquid ratio, the quadratic effect of ethanol concentration, the linear effect of temperature and the effect quadratic of the solid–liquid ratio. Likewise, the ethanol concentration favored recovery of antioxidant compounds (Figure 2b). Additionally, it is important to highlight that the solid–liquid ratio had a higher influence on this response, when compared to other responses evaluated in this study.

The antioxidant capacity measured by FRAP assay was dependent on ethanol concentration and extraction temperature, being observed to have a negative effect of ethanol concentration and positive effect of temperature. From the response surface, it is possible to note that this potential is higher when the temperature used in the extraction process was superior to 60 °C and ethanol concentration was between 10 and 50% (Figure 2c).

Therefore, the influence of ethanol concentration (<50%) and temperature (>60 °C) on recovery of bioactive compounds of umbu fruit peel is highlighted. The use of a binary solvent containing more water than ethanol was found to be more efficient for extraction. Ribeiro et al. [14] also reported that using 30% ethanol in water as an extractive solution was more efficient to extract antioxidant compounds of siriguela peels (*Spondias purpurea*). Jesus et al. [15] published a positive effect of binary solvent on recovery of antioxidant compounds of vine pruning residues. According to those authors, mixtures of alcohols/water were more efficient in the extraction of phenolic compounds than mono-component solvent due to the increase in membrane permeability of the plant material. Additionally, Oreopoulou et al. [16] reported that the efficiency of a solvent depends mainly on its ability to extract bioactive compounds, where ethanol is an adequate solvent to solubilize flavonoid glycosides, while water become more able dissolve phenolic acid glycosides, corroborating the use of a binary solvent system composed by ethanol and water. In addition, ethanol is a green, abundant and non-toxic solvent, which increases its use in the extraction processes.

The extraction temperature also has an important role in the extraction of bioactive compounds, since it increases both solute solubility and the diffusion coefficient of phenolic compounds as reported by Ruíz-García et al. [17], who obtained a higher content of phenolic compounds from grape skin when the extraction temperature was increased from 23 °C to 57 °C. However, it is important to emphasize that our results reveal that the positive effect of temperature was accompanied by the effect of the ethanol percentage in the extraction solution, which corroborates the optimization of extraction processes. Furthermore, as reported by Markom et al. [18], the surface tension and viscosity of the solvent are drastically reduced at boiling point when compared to at a lower temperature. In this context, the solvent can easily reach the cell wall of the plant material. Thus, the bioactive-rich extract is mainly the result of the synergistic effect of the ethanol concentration in the extractive solution and the process temperature.

### 2.2. Selection of the Optimal Operational Condition

**Figure 3:**
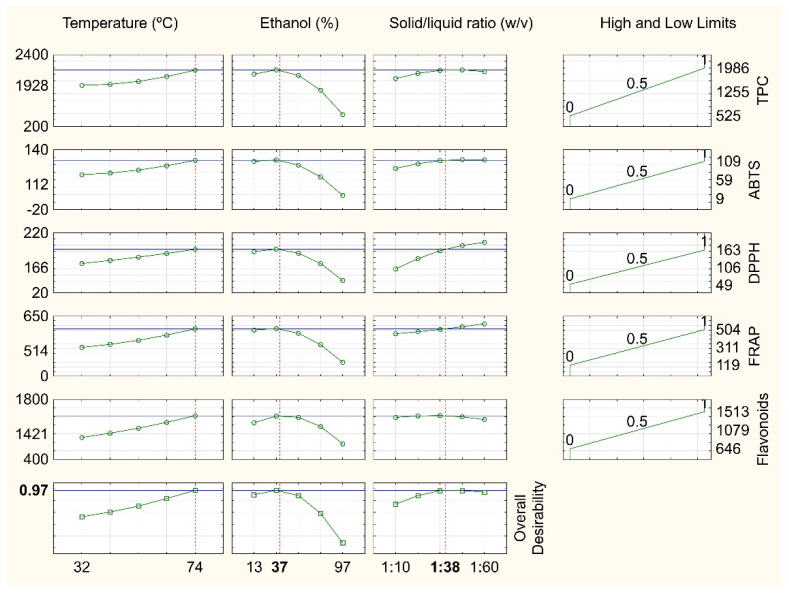
Profile of predicted values for individual and overall desirability for the extraction optimization. TPC—total phenolic compounds (mg GAE/100 g); TFC—total flavonoid compounds (mg RE/100 g); ATBS^•+^—(μmol TE/g); DPPH^•^—(μmol TE/g); FRAP—(μmol Fe^2+^/g).

As cited above, each response presented different operational conditions for the extraction of bioactive compounds of umbu fruit peel. Thus, to better understand the results and to obtain the optimal condition of temperature, ethanol concentration and solid–liquid ratio, which would improve the recovery of bioactive compounds, the simultaneous optimization method was used. Figure 3 shows individual and overall desirability profiles for the extraction conditions and evaluated responses. The overall desirability value reached was equal to 0.97, corresponding to the optimal operational condition for extraction of bioactive compounds from fruit peel. Therefore, the extraction should be performed at 74 °C, using ethanol 37%, and a solid–liquid ratio of 1:38 in order to maximize the recovery of these compounds.

In this operational condition, observed values of TPC (1985 mg GAE/100 g); TFC (1364 mg RE/100 g); and antioxidant capacity by ABTS^•+^ (122 µmol TE/g), DPPH^•^ (174 µmol/TE g), and FRAP assays (468 µmol Fe^2+^/g) were close to the predicted values by the experimental design as follows: TPC (1928 mg GAE/100 g); TFC (1421 mg RE/100 g); antioxidant capacity by ABTS^•+^ (112 µmol TE/g), DPPH^•^ (166 µmol TE/g), and FRAP assays (514 µmol Fe^2+^/g) with coefficients of variation less than 7%. Therefore, the results showed that the experimental design is adequate to obtain the optimal operational condition for extraction of bioactive compounds of umbu fruit peel.

### 2.3. Bioactive Profile by LC-HRMS

LC-HRMS analysis allowed the identification of 15 different chemical compounds in the extract (Table 2 and Table 3). In accordance with experimental section, identification was performed by MS/MS fragmentation pattern, comparison with the GNPS library and selection of those hits that were previously isolated from *Spondias* spp. [3,19,20], compounds from species belonging to the Anacardiaceae family or compounds common in plant species (see Table 2 and Table 3). It was not possible to construct clusters due to the relatively low fragmentation pattern of the sample. Compounds were better ionized in positive mode. Pipecolic acid and anthranilic acid and their derivatives were proposed since they are very common plant secondary metabolites, including in fruits [21,22]. The annotation of compounds 2’-hydroxy-4’-methoxyacetophenone, 4-acetyl-2-prenylphenol, and rubinaphthin A was based on phenolic compounds that can be found in many plant species, as well as phenolic acids that have already been identified in *Spondias* spp., such as gallic acid, 3,5-dihydroxybenzoic acid, and coumaric acid [3,19,20]. That noted, flavonoids, benzoic acid derivatives, glycosides (koaburside), C_6_-C_3_ derivatives, among other compounds, were identified in the extract. Some attention should be paid to rutin, which was identified in both ionization modes. This compound was previously identified in umbu fruit and has shown many biological properties such as cardiovascular, anticancer, anti-inflammatory, antidiabetic, anti-obesity, antimicrobial, anti-leishmanial, antioxidant activities and others [3,19,20,23]. It is already known that flavonoids and phenolic acids have great antioxidant properties, as shown in our work [24,25].

### 2.4. Antimicrobial Assays

The antimicrobial action of the optimized extract from umbu fruit peel was tested against a variety of both Gram-positive and Gram-negative bacteria as well as against *Candida* species (Table 4). The results showed a distinct ability of the umbu fruit peel extract to inhibit the microbial viability with more action on Gram-positive bacteria (MIC values varying from 0.03 to 0.06 mg GAE/mL) compared to Gram-negative (MIC = 0.12 mg GAE/mL), while it was completely ineffective against *Candida* species, which reveals its ability to act against bacteria, but not against fungi. The minimum bactericidal concentration ranged from 0.06 to 0.24 mg GAE/mL, making the Gram-positive more susceptible than Gram-negative bacteria. These results can be explained, at least in part, due to the morphological differences observed between these two groups, in which Gram-negative bacteria have an extra outer membrane together with periplasmic space that serves as a selective permeation barrier, thus reducing chemical interaction and inhibition effects of extract [32]. Various studies have attributed the inhibitory effect of plant extracts against different bacteria to their phenolic compounds, such as those tentatively identified in the present work by LC-HRMS (Table 2 and Table 3). These compounds can present the ability to bind with the bacterial cell wall and then inhibit the bacterial growth. Additionally, phenolic compounds may precipitate protein and inhibit enzymes of microorganisms [33]. Moreover, it is relevant to stress that antibacterial action may be due to the synergy of several compounds, including phenolic acids and flavonoid derivatives and other bioactive compounds presented in Table 2 and Table 3.

### 2.5. α-Amylase Inhibition

In this set of experiments, the effect of extract from umbu fruit peel on α-amylase activity was evaluated. The results revealed that umbu extract inhibited the α-amylase activity in a typically dose-dependent way. In this context, the extract at a concentration of 0.01 mg GAE/mL showed a percentage of inhibition of 38.3% and at 0.273 mg GAE/mL it was increased to 87.4%. The extract presented an IC_50_ value of 0.076 mg GAE/mL. The acarbose is widely used in medicine as an inhibitor of digestive enzymes related to the breakout of polysaccharides. As these enzymes are inhibited, there is reduction in the absorption of glucose, and consequently the reduction of postprandial blood glucose level elevation, which helps to reduce risk of Diabetes Mellitus and other diseases [34,35]. The IC_50_ value of the standard drug was found to be 0.034 mg/mL. Even though a lower concentration of this medicine is required for inhibition at 50% α-amylase activity when compared to the umbu fruit extract, it is highlighted that this extract, obtained from residue of umbu fruit depulp, presented good inhibitory activity against α-amylase when compared to literature data. Laaroussi et al. [36] reported that different propolis samples from Morocco presented IC_50_ values between 0.195 and 0.964 mg/mL, being, therefore, higher than that found in umbu fruit extract. This comparison is interesting, because the phytochemical composition of propolis samples indicated the presence of phenolic acids, flavonoids and stilbenes, which is similar to umbu fruit extract composition. Thus, these results indicate that umbu fruit extract is a promising candidate for control and prevention of Diabetes type 2.

## 3. Materials and Methods

### 3.1. Umbu Fruit Peel

For this work, ripe umbu fruits (2.4 kg) purchased on the local market of Rio de Janeiro were used. For that, they were sanitized using sodium hypochlorite (100 ppm) and manually depulped using a domestic sieve. Then, umbu peels were dried in an oven with forced air circulation at 45 °C for 45 h. The dried peels were disintegrated in a domestic mixer to obtain a powder. It was presented with moisture equal to 17% (*w*/*w*), gravimetrically determined at 105 °C.

### 3.2. Thermal-Assisted Solid–Liquid Extraction

The extraction of bioactive compounds was performed by agitated solvent extraction, using 125 mL glass flasks duly covered and heated for 60 min under constant stirring of 130 rpm. These variables were fixed according to Ribeiro et al. [14], who obtained antioxidant compound-rich extract from siriguela peels. The work temperature was selected taking into account the boiling point of ethanol in order to avoid loss during the extraction process. The extraction temperature, ethanol percentage and solid–liquid ratio (*w/v*) were ranged aiming to evaluate their effect on responses. The variation interval of the independent variables was selected based on preliminary data and works published by our laboratory [14,37]. Obtained extracts were filtered in quantitative filter paper (FP41, Quanty) and stored in the freezer until further analysis.

### 3.3. Experimental Design

The effect of the independent variables (extraction temperature, ethanol percentage in the extractive solution and solid–liquid ratio) on the content of total phenolic compounds (TPC) and total flavonoid compounds (TFC) and antioxidant capacity by ABTS^•+^, DPPH^•^ and FRAP assays were evaluated using the response surface methodology (RSM) based on rotational central composite design, composed of 8 factorial points (level ±1), 3 central points (level 0) and 6 axial points (level ±1.68), resulting in 17 trials. Table 1 shows the combination of the independent variables (coded and real values). The experimental data were analyzed by RSM, using the second order polynomial equation. Analysis of variance (ANOVA), test for the lack of fit and coefficient of determination (R^2^) were used to verify model significance.

To determine the optimal condition for extraction of the bioactive compounds of umbu fruit peel, the technique of simultaneous optimization of independent variables (desirability) was used. The desirability function is based on the conversion of each response in an individual desirability (d). After that, they are combined into an overall desirability (*D*), using the geometric mean. The *D* value ranges from zero (0) to one (1), in which the value of 1 corresponds to the desirable response [38]. Under the optimal operational condition, more assays were performed and observed results were compared with those predicted by the model.

### 3.4. Chemical Analysis

#### 3.4.1. Total Phenolic Compounds (TPC)

This determination was performed using the Folin–Ciocalteu reagent (Imbralab, Ribeirão Preto, Brazil) according to the method described by Georgé et al. [39]. For the reactions, 250 μL of each filtered and properly diluted extract were mixed with 1250 μL of 10% (*v*/*v*) Folin–Ciocalteu reagent and 1000 μL of 7.5% (*w/v*) Na_2_CO_3_ solution. Thereafter, samples were heated at 50 °C for 15 min and cooled at room temperature using an ice bath. The absorbance was measured at 760 nm. A calibration curve was created from a gallic acid standard, which ranged from 10 to 100 mg/L. TPC content was expressed as mg gallic acid equivalent per 100 g (mg GAE 100/g).

#### 3.4.2. Total Flavonoid Compounds (TFC)

The TFC content was determined based on the method described by Zhishen et al. [40] with minor modifications. Here, 0.5 mL of extract was mixed with 3.2 mL of ultrapure water and 150 μL of NaNO_2_ (5%). After homogenization, the mixture was left to stand for 5 min. Thereafter, 150 μL of AlCl_3_ (10%) was added to the mixture, and 1 mL of NaOH (1 M) was added after one minute. The absorbance was recorded at 510 nm with a spectrophotometer (Metash, Shanghai, China) using ultrapure water as a blank. The TFC content was calculated using the calibration curve of rutin, with concentration ranging from 99 to 595 mg/L. The results were expressed as mg rutin equivalents per 100 g (RE/100 g).

#### 3.4.3. ABTS^•+^ Assay

The antioxidant capacity was determined by the reduction method of the ABTS^•+^ radical (Sigma-Aldrich, Saint Louis, MO, USA) according to Gião et al. [41]. For the reactions, 30 μL of each filtered and duly diluted extract were mixed with 3000 μL ABTS^•+^ radical. After 6 min, the absorbance was measured at 734 nm with a spectrophotometer in spectrophotometric units (Metash, Shanghai, China) using ultra-pure water as a blank. The ABTS^•+^ antiradical activity was calculated using Trolox solutions (Sigma-Aldrich, Buchs, Switzerland) with different concentrations in a range of 500–2000 μmol. Results were expressed as µmol Trolox equivalents per gram (μmol TE/g).

#### 3.4.4. DPPH^•^ Assay

The DPPH^•^ radical (Sigma-Aldrich, Steinheim, Germany) scavenging activity of extracts was determined according to the method described by Hidalgo, Sánchez-Moreno and Pascual-Teresa [42]. For the reactions, 100 μL of each duly diluted extract was added to 2900 μL of DPPH^•^ solution (6×10^−5^ M in methanol and diluted to an absorbance of 0.700 at 517 nm). The resulting solutions were allowed to stand for 30 min in darkness at room temperature. After that, the absorbance was measured at 517 nm with a spectrophotometer (Metash, Shanghai, China) using methanol as blank. The DPPH^•^ radical scavenging activity was calculated using Trolox solutions with different concentrations in a range of 80–700 μmol. Results were expressed as µmol Trolox (Sigma-Aldrich, Buchs, Switzerland) equivalents per gram (μmol TE/g).

#### 3.4.5. FRAP Assay

This assay was performed according to Benzie and Strain [43] with slight modifications. Stock solutions included 300 mM of acetate buffer (pH 3.6), 10 mM TPTZ (Sigma-Aldrich, Buchs, Switzerland) in 40 mM HCl and 20 mM FeCl_3_·6H_2_O. The work solution was prepared by mixing 25 mL of the acetate buffer, 2.5 mL of TPTZ solution and 2.5 mL of FeCl_3_·6H_2_O. 100 μL of each extract was reacted with 3 mL of FRAP at 37 °C for 30 min. The absorbance was measured at 593 nm. The ferric reducing ability power was calculated using FeSO_4_·7H_2_O solutions with different concentrations in a range of 150–1200 μmol. The results were expressed as μmol Fe^2+^/g.

#### 3.4.6. UPLC-qTOF/MS Analysis

Sample extract was dissolved in an aqueous solution of formic acid (0.1%, *v*/*v*) and subjected to Ultra-performance liquid chromatography coupled to quadrupole/time-of-flight mass spectrometry (UPLC-qTOF/MS) system from Bruker Daltonics (MaxisImpact, QTOf Bruker, Bremen, Germany). The sample separation was conducted on a Hypersil C18 column (3 µm particle size, 2.1 mm i.d. × 150 mm). The column temperature was maintained at 40 °C. An aliquot of 20 μL of the extract solution at 100 ppm was injected on equipment under flow rate of 0.27 mL/min. Linear gradient elution of A (0.1% formic acid in water) and B (acetonitrile) was applied with the following gradient: 5% B, and then linearly increased to 9% B within 5 min, then 9% B was increased to 16% within 10 min, 16% B increased to 36% B within 18 min, and 36% B increased to 95% B within 1 min, then holding in this concentration for 12 min. Next, 95% B was decreased to 5% B within 1 min, and finally held in this way for 13 min. Data Analysis (Bruker Daltonics, Bremen, Germany) was used for data interpretation. The mass spectra (MS) were acquired in both negative and positive modes with an electrospray ionization source (ESI). The data were scanned for each test sample from 50 to 1200 *m/z*. Highly purified nitrogen (N_2_) was used as the nebulizing gas and ultra-high pure helium (He) as the collision gas. In terms of negative electrospray mode, the capillary voltage was set at 5000 V. ESI parameters applied were: dry gas: 200 °C; dry gas flow: 8 L/min; nebuliser: 2 bar. The acquired data were converted to mzML format using MSConvert software (http://proteowizard.sourceforge.net/ (accessed on 10 December 2021)) and submitted to Global Natural Products Social Molecular Network (GNPS [44]; http://gnps.ucsd.edu (accessed on 10 December 2021)) online system. The molecular network calculations and database matching were constructed using 2.0 Da as precursor ion mass tolerance and 0.05 Da as fragment ion mass tolerance, 0.7 as minimum cosine score and 3 as minimum matched fragment ions for edge linkage. Finally, GNPS data were then imported and visualized using the Cystoscope software (version 3.8.0) to find the subnetworks portions.

### 3.5. In Vitro Biological Studies

Microorganisms and culture conditions Gram-negative (*Acinetobacter baumannii* ATCC 19606, *Escherichia coli* ATCC 25922, *Klebsiella pneumoniae* ATCC 13883, and *Pseudomonas aeruginosa* ATCC 27853) and Gram-positive (*Staphylococcus aureus* ATCC 29213, *Staphylococcus epidermidis* ATCC 12228 and *Bacillus subtilis* 168 LMD 74.6) bacteria were grown in Mueller–Hinton agar (Difco, Franklin Lakes, NJ, USA) for 24 h at 35 ± 2 °C. The yeasts *Candida albicans* ATCC 90028 and *Candida tropicalis* ATCC 750 were cultured in Sabouraud dextrose agar (Difco, Franklin Lakes, NJ, USA) for 24 h at 35 ± 2 °C.

#### 3.5.1. Antimicrobial Assays

For these assays, the optimized extract was evaporated in a rotavapor under reduced pressure to eliminate ethanol. After that, the concentrated optimized extract presented TPC content equal to 0.965 mg GAE/mL. Antimicrobial activity was evaluated using the broth microdilution method in 96-well polystyrene plates, standardized according to document M07-A9 (for bacterial assays) and M27-A3 (for fungal assays). The minimum inhibitory concentration (MIC) was determined by visual inspection after incubation at 37 °C for 24 h of extracts at final concentrations of 0.03, 0.06, 0.12 and 0.24 mg GAE/mL. To determine the minimum bactericidal and fungicidal concentration (MBC and MFC), 10 μL of the wells that had no visible microbial growth were inoculated in Mueller–Hinton culture medium and Sabouraud Dextrose Agar for 24 h at 37 °C. The MBC and MFC were considered to be the lowest concentration capable of completely inhibiting microbial growth on the agar surface.

#### 3.5.2. Assay for α-Amylase Inhibition

The inhibition assay for α-amylase was performed as reported by Meng et al. [45] with minor modifications. Briefly, 100 μL of extract evaporated in a rotavapor under reduced pressure to eliminate ethanol at different dilutions, was mixed with α-amylase solution (100 μL, 1.0 U/mL) (Sigma-Aldrich, St. Louis, MO, USA) in phosphate buffer (pH 6.9) and 250 μL of 1% starch solution. The incubation was carried out for 5 min at 37 °C. The enzyme reaction was stopped by adding dinitrosalicylic acid reagent (Sigma-Aldrich, Steinheim, Germany) (250 μL) and incubation was carried out for 15 min in boiling water. For dilution, 2 mL distilled water was added to the final reaction mixture. The absorbance was read at 540 nm. The inhibitory effect was calculated by Equation (1). The results were expressed as IC_50_ (mg GAE/mL). Acarbose (Supelco, Laramie, WY, USA) was used as positive control in order to compare the inhibitory effects.

Inhibition percentage (%) = [1 − (Abs_sample_ − Abs_control-1_)/Abs_control-2_] × 100 (1)

where the Abs_control-1_ is the result of reaction without adding enzyme, which was replaced for buffer solution, while the mixture of enzyme and starch solution without extract was Abs_control-2_.

### 3.6. Statistical Analysis

All measurements were performed in triplicate, and the results were analyzed using Statistic 13 software (Dell Inc.) [46]. The experimental design data were analyzed by RSM, using the second order polynomial equation. Analysis of variance (ANOVA), test for the lack of fit and coefficient of determination (R^2^) were used to verify model significance. The desirability function was applied to determine the operational parameters of extraction that could improve the recovery of bioactive compounds from umbu fruit peel. A 5% level of significance was employed for all analysis.

## 4. Conclusions

The recovery of bioactive compounds from umbu fruit peel was mainly affected by the ethanol percentage of the extractive solution and extraction temperature. Less apolar binary solvent systems and high temperature provided extracts rich in bioactive compounds. The optimal operational conditions to recover these compounds were 74 °C, 37% ethanol as solvent, and a solid–liquid ratio of 1:38. Fifteen compounds were identified in the optimized extract, which mainly comprised phenolic acids and flavonoids. This extract showed antioxidant and antimicrobial activities, particularly antibacterial action, and it was able to inhibit α-amylase enzyme. Thus, this study allowed the identification of optimal operational conditions to obtain a bioactive-rich extract from umbu fruit peel, a residue of processing of this native fruit of Brazil.

## Figures and Tables

**Table 1:** Real and coded values of the independent variables employed to recover the bioactive compounds from umbu peels and total phenolic (TPC) and flavonoids (TFC) compounds and antioxidant capacity values of the extracts.

Trials	Temperature	Ethanol	Solid–Liquid Ratio	TPC ^1^	TFC ^2^	ABTS^•+ 3^	DPPH^• 3^	FRAP ^4^
	(°C)	(%)	(g/mL)					
1	40 (−1)	30 (−1)	1:20 (−1)	1280	925	74	95	319
2	40 (−1)	30 (−1)	1:50 (+1)	1644	1015	83	136	364
3	40 (−1)	80 (+1)	1:20 (−1)	603	692	25	49	150
4	40 (−1)	80 (+1)	1:50 (+1)	811	700	25	82	130
5	65(+1)	30 (−1)	1:20 (−1)	1593	1203	88	113	443
6	65 (+1)	30 (−1)	1:50 (+1)	1677	1207	101	163	448
7	65 (+1)	80 (+1)	1:20 (−1)	731	867	34	65	180
8	65 (+1)	80 (+1)	1:50 (+1)	850	877	37	96	246
9	32 (−1.68)	55 (0)	1:35 (0)	1231	847	58	105	289
10	74 (+1.68)	55 (0)	1:35 (0)	1986	1513	109	162	504
11	53 (0)	13 (−1.68)	1:35 (0)	1315	906	74	126	348
12	53 (0)	97 (+1.68)	1:35 (0)	525	646	9	51	119
13	53 (0)	55 (0)	1:10 (−1.68)	1075	1121	61	71	321
14	53 (0)	55 (0)	1:60 (+1.68)	1652	1038	74	160	442
15 (CP)	53 (0)	55 (0)	1:35 (0)	1479	1055	73	121	316
16 (CP)	53 (0)	55 (0)	1:35 (0)	1379	1087	72	125	346
17 (CP)	53 (0)	55 (0)	1:35 (0)	1405	1186	77	128	364

CP—Central point. ¹ Results expressed as mg GAE/100 g. ^2^ Results expressed as mg RE/100 g. ^3^ Results expressed as µmol Trolox/g. ^4^ Results expressed as µmol Fe²^+^/g.

**Table 2:** Metabolites tentatively identified by LC-HRMS in negative ion mode.

#	*t*_R_ (min)	*m/z* Observed	*m/z* Theoretical	Molecular Formula	Fragment Ions (*m/z*)	Metabolite	Organism/Reference
**1**	4.14	153.0200	153.0193	C_7_H_6_O_4_	125.0261; 109.0279	3,5-Dihydroxybenzoic acid	Already described in *Spondias* spp. [19]
**2**	18.45	609.1482	609.1461	C_27_H_30_O_16_	301.0357; 300.0288; 273.0350; 257.0430; 151.0033	Rutin	Already described in *Spondias* spp. [3,20]
**3**	18.72	463.0862	463.0882	C_21_H_20_O_12_	300.0256; 271.0236; 255.0342	Isoquercitrin	Already described in *Spondias* spp. [19]
**4**	20.62	593.1535	593.1512	C_27_H_30_O_15_	285.0372; 284.0343; 257.0501; 255.0366; 227.0402	Kaempferol 3-*O*-rutinoside	Already described in *Spondias* spp. [19]
**5**	35.27	193.0709	193.0506	C_10_H_10_O_4_	178.0512; 149.0979; 134.0676	Ferulic acid	Already described in *Spondias* spp. [19]

**Table 3:** Metabolites tentatively identified by LC-HRMS in positive ion mode.

#	*t*_R_ (min)	*m/z* Observed	*m/z* Theoretical	Molecular Formula	Adduct	Fragment Ions (*m/z*)	Metabolite	Organism/Reference
**6**	1.58	325.1329	325.1129	C_12_H_22_O_11_	[M − H_2_O + H]^+^	145.0502; 127.0399; 85.0297; 69.0342; 55.0188	Sucrose	Very common in plants
**7**	3.10	130.0863	130.0863	C_6_H_11_NO_2_	[M + H]^+^	84.0427; 57.0692; 56.0506	Pipecolic acid	Found in *Citrus* spp. [21]
**8**	7.47	165.0545	165.0546	C_9_H_8_O_3_	[M + H]^+^	147.0445; 120.0824; 119.0515	Coumaric acid	Already described in *Spondias* spp. [3,20]
**9**	7.82	347.1670	347.1337	C_15_H_22_O_9_	[M + H]^+^	185.0790; 154.0640; 153.0560; 125.0600	3,4,5-Trimethoxyphenyl beta-D-glucopyranoside (Koaburside)	Found in *Rhus parviflora* (Anacardiaceae) [26]
								Found in *Cladogynos orientalis* (Euphorbiaceae) [27]
**10**	9.62	138.0557	138.0550	C_7_H_7_NO_2_	[M + H]^+^	121.0657; 92.9800; 65.0410	Anthranilic acid	Found in *Arabidopsis thaliana* (Cruciferae) [28]
**2**	18.40	611.1614	611.1607	C_27_H_30_O_16_	[M + H]^+^	465.1022; 303.0496; 145.0511; 129.0568	Rutin	Already described in *Spondias* spp. [3,20]
**3**	18.54	465.1028	465.1028	C_21_H_20_O_12_	[M + H]^+^	447.1002; 303.0463; 258.0178; 231.1018	Isoquercitrin	Already described in *Spondias* spp. [19]
**11**	19.42	167.0705	167.0703	C_9_H_10_O_3_	[M + H]^+^	149.0260; 125.0960; 121.0310	2’-Hydroxy-4’-methoxyacetophenone (Paeonol)	found in *Paeonia* spp. (Ranunculaceae) [29]
**12**	36.01	205.1166	205.1223	C_13_H_16_O_2_	[M + H]^+^	149.0255; 121.0309; 107.0825; 59.0501	4-Acetyl-2-prenylphenol	Found in *Polymnia sonchifolia* (Asteraceae) [30]
**13**	36.11	581.1551	581.1501	C_26_H_28_O_15_	[M + H]^+^	303.1460; 302.1490; 153.0967; 149.0236	Quercetin-deoxyhexosyl-pentoside	Very common in plants
**14**	38.09	389.2336	389.0843	C_17_H_18_O_9_	[M+Na]^+^	149.0240; 147.0656; 129.0550; 71.0850; 57.0705	Rubinaphthin A	Found in *Rubia* spp. (Rubiaceae), i.e., *Rubia yunnanensis* [31]
**15**	42.19	197.0812	197.0808	C_10_H_12_O_4_	[M + H]^+^	179.0861; 169.0027; 137.0633; 95.0850	Dihydroferulic acid	Very common in plants

**Table 4:** Antimicrobial activity of umbu fruit peel extract.

Microorganisms	Antimicrobial Assays (mg GAE/mL) ¹	
	MIC Values	MBC/MFC Values
**Gram-positive bacteria**		
*Bacillus subtilis* 168 LMD 74.6	0.06	0.12
*Staphylococcus aureus* ATCC 29213	0.06	0.06
*Staphylococcus epidermidis* ATCC 12228	0.03	0.12
**Gram-negative bacteria**		
*Escherichia coli* ATCC 25922	0.12	0.24
*Acinetobacter baumannii* ATCC 19606	0.12	0.24
*Psedomonas aeruginosa* ATCC 27853	0.12	0.24
*Klebsiella pneumoniae* ATCC13883	0.12	0.24
**Fungi**		
*Candida albicans* ATCC 90028	ND	ND
*Candida tropicalis* ATCC 750	ND	ND

ND—not detected. ¹ Results expressed as mg gallic acid equivalent/mL. MIC—minimum inhibitory concentration. MBC—minimum bactericidal concentration. MFC—minimum fungicidal concentration.

## Data Availability

Data is contained within the article.
